# Physiology

**Published:** 1826-01

**Authors:** 


### Quarterly Periscope

OF

### PRACTICAL MEDICINE;

BEING

### The Spirit of the Medical Journals,

*Foreign and Domestic;*

WITH COMMENTARIES.

### I.

### Physiology.

——————— latas à numine leges
Religiosa doce, mentesque cupidine veri
Allice.

1. *Disorganization of the Medulla Oblongata, without Disturbance of the Nervous Functions.* By A. Velpeau, M.D. *(Archives Generales.)*

The pleasure resulting from the acquisition of knowledge is not uniformly without alloy. The march of science destroys many a happy dream which had long been enjoyed without question—many a proud theory which had been deemed invulnerable—many a darling prejudice which had become, as it were, a part of our nature, and which we cannot tear away from our minds without a painful struggle. But this is not the worst. Facts are every now and then turning up which fly in the teeth of *all known laws of the animal economy*, and almost turn science into ridicule! No wonder that so much uncertainty should prevail in the laws which govern morbific and remedial agencies on the animal economy, when the most positive and fundamental laws of *physiology* are occasionally crossed, not merely by anomalies, but by downright contradictions! By what physiological law do sense and motion continue unimpaired in the body after the medulla oblongata is destroyed? This question is more easily asked than answered. But the difficulties which it involves ought to teach humility to those who are intoxicated with the pride of science, and induce medical men to be less positive in their own opinions and experience, and more charitable towards the opinions and experience of their neighbours, on other points still more liable to deception.

*Case* 1. A young lad of 18 years, delicate, but healthy for some years previously, struck his head rather violently against a mantle-piece, while rising from his seat, in the month of November, 1823. The pain was not great, and only a slight contusion of the scalp, over the occipital bone, was at first perceived. Two months afterwards a swelling appeared on the part, from which, when opened, there issued some ill-conditioned matter. When a probe was introduced, the surgeon found that the scalp was detached from the pericranium for some distance around the suppuration. This last continued abundant. The cervical glands swelled, and the least motion of the head was accompanied by severe pain. Pain was also constant in the back part of the head and neck. On the 15th March, 1824, the wound was enlarged, but no mitigation of pain resulted. On the 30th, he entered the Hospital of St. Como. His face was now pallid—livor round the eyes—lips blueish—tongue moist—slight fever—skin dry and hot. In the centre of the wound was seen a piece of naked bone, the size of a two-franc piece. The pus, however, was good. The least attempt to turn the head was attended with insupportable pain. There were also dull pains constantly in the occipito-vertebral region. In the evenings there was œdema of the lower extremities—the urine was scanty and sedimentous. The patient could walk about, though he complained of being very weak. We pass over the details between this and the 11th of April, when the necrosed piece of bone was found to be moveable, with a hole in its centre, through which could be distinguished the motions of the brain. Symptoms of gastro-enteritis were now added to the intolerable sufferings about the head and neck, and he lingered out till the 17th of April, when death closed the scene.

*Dissection.* The necrosed portion of the occipital bone was circular, with a hole in its centre. The surrounding pericranium was sound. The dura mater corresponding to this portion was thickened and discoloured. With these exceptions all the encephalic organs appeared healthy, till they came to the medulla oblongata, which, to their astonishment, was separated from the medulla spinalis—at least there was only a kind of gellatinous bouillie (espèce de bouillie diffluente) which connected them. The nervus accessorius and hypoglossus traversed this disorganized portion without damage:—the par vagum, on the contrary, and the glosso-pharyngeus of the left side, were completely severed, and their roots were no where visible. Under, and concealed by this diseased portion of medulla oblongata, was found the odontoid process of the second vertebra (dentata) bare and pressing on the said diseased portion.

There is a long account of diseased appearances, which we need not any farther pursue in this place. The whole forms a very remarkable exception to the general laws of the nervous system, and can only be considered as an anomaly not very likely to happen often.

There is but one way of accounting for the above phenomena that has any thing of feasibility in it. It is this:—that the nervous influence may have been carried through the disorganized and semifluid portion of medulla oblongata, as the electric fluid is carried from one extremity of a divided nerve to the other, when left nearly in contact, or with any conducting substance intervening. This was the case, as is well known, in some of Dr. Philip's experiments on the rabbits, at the Royal Institution, and which, for some time, puzzled the experimenters. If this be the case, pathology has, in the instance cited, come to the elucidation of physiology.

In a subsequent Number of the same Journal, (for March, 1825,) M. Velpeau returns to the subject, and relates some very curious cases that occurred in the public practice of Professor Bougou, in *La Clinique externe de la Faculté.* Of these we shall take some notice.

*Case* 2. J. Chardonnens, aged 17, presented himself at the Clinique on the 17th of May, 1823, for a disease of the spine, and an ulcer on the left thigh. This youth had been rickety from his infancy. About the age of 8 he had fixed pains in the loins, and then the spinal column began to form a curvature. At a later period he had an abscess in the thigh, which discharged much matter and remained fistulous. As the patient had no loss of power or of sensibility in the lower extremities, and as the sore in the thigh did not seem to have any connexion with the spinal curvature, he was suspected to have a syphilitic taint, and the oxymuriate of mercury was given. In a month all the symptoms became aggravated. Several lumbrici were discharged from the ulcer, together with a large quantity of black and fetid matter. This discharge ceased all at once without known cause, and presently the patient began to complain of pain in the right hypochondrium, difficulty of breathing, and fever. These symptoms were checked a little by antiphlogistic measures, but hectic fever came on, and the patient died on the 11th of June, 1823.

*Dissection.* The arachnoid was much thickened, and several portions of the brain were softened. Nothing else remarkable in the head. The right kidney was enlarged, and the left converted into a cyst, filled with a fluid resembling urine. Behind the peritoneum, and in the lumbar and iliac regions, as well as in the pelvis, was found a large quantity of black purulent matter, mixed with the detritus of different tissues. The cavities formed in these parts communicated, by a passage under the crural arch, with the ulcer in the thigh. The bones of the spine were slightly carious, from the 10th dorsal vertebra to the extremity of the sacrum. The bodies of the three lumbar vertebræ, and a portion of the upper part of the sacrum had entirely disappeared, and their spinal processes had become firmly ossified. At this point the spinal column made a sharp angle or projection forward—and here the spinal marrow was almost entirely destroyed, being rotten, soft, and many of the nerves, going to the lower extremities, separated from their origins. Nearly four inches of the dura matral covering of the spinal marrow at this place were wanting.

*Case* 3. In the month of July, 1823, there died, in the same hospital, a man about 30 years of age, who had been affected with phthisis, and had also a curvature (forward) of the spine. The latter disease he made little mention of. On being interrogated, while in the hospital, he said that he had, for two or three years, suffered pain in his back; but he always made light of this, and recurred to his principal complaint, pains in the chest and distressing cough. Four days before his death he got out of bed, and exhibited no symptom of paralysis or want of feeling in any of his limbs.

On dissection, besides the usual tuberculated state of the lungs, there was found a remarkable disease of the whole spine. All the dorsal vertebræ were carious in their bodies, as well as the sacrum and coccyx. The caries was superficial in the upper vertebræ, but became deeper and deeper as it descended towards the sacrum, so that of the lower dorsal and all the lumbar vertebræ there were only mere fragments of bone left. There was a space of more than four inches filled with a reddish fluid matter instead of spinal marrow.* It was only by means of the spinous and transverse processes that the body was kept erect, or the continuity of the spinal column preserved.

This case is certainly a puzzler for the physiologist. It made such an impression on our author, that he began to look about for other instances of an analogous nature; and he has been able to find a considerable number on record. We cannot afford space for notice of all the cases he has collected, but some of them are worthy of a place here.

*Case* 4. In the Academy of Sciences is preserved a preparation where the spinal marrow had been cut across by the point of a sword, which broke and remained jammed in the vertebral column. The man marched 80 leagues before his death. He presented himself at the Hopital de Niort, where, on examination, there was found an abscess on the back. This being opened and the matter evacuated, the piece of sword was discovered and extracted. The patient died in 36 hours afterwards, and the above facts were ascertained. (*Academie de Sciences, p.* 123.)

*Case* 5. A soldier died at the Hotel Dieu, 24 hours after being wounded by a musket ball, with all the symptoms of effusion in the chest. A few minutes before his death he made violent efforts with his lower extremities, so as to do away with all idea of paralysis. Yet, on dissection, it was found by Desault himself (who records the case in his own journal) that the ball had completely divided the spinal marrow opposite the tenth dorsal vertebra. (*Desault's Journal, vol.* IV. *p.* 137.)

*Case* 6, M. Janson, of Lyons, found in the body of a distorted girl,

* "Pour la moelle elle-même, il n'y en avait pas vestige dans l'espace abandonné par les vertebres. Cet espace etait remplis par la matière rougeâtre que l'indiquais tout a l'heure."—331. The preparation is preserved in the Museum of the Faculty.

13 years of age, two of the dorsal vertebræ destroyed by caries—the spinal marrow flattened for the space of five inches, and converted into a soft and diffluent pulp, while in one place it was entirely wanting for the space of five or six lines. This young girl had been seen walking about a few days before her death, without any particular diminution of the interior functions.

*Case 7.* M. Ollivier reports the case of a boy who lived to the age of eight years. The dorsal vertebræ were carious, and four inches of the spinal marrow were entirely wanting. The investing membranes, however, remained.

Our author goes on to cite 25 cases, including the famous one published by Magendie, and of which we gave an account at the time. From these cases, he observes, and numerous others that might be collected, we are almost unavoidably forced to conclude, that the spinal marrow is not indispensable to sense and motion in animals, including man himself—or, at least, that it is not the only and exclusive organ for these purposes. In short, from these premises it would seem probable that the nervous trunks themselves may, in some instances, have the power of supplying the muscles with moving power, and the surface with sensibility, independently of the brain and spinal marrow, of which they are considered as mere offsets. But we leave these questions to the professed physiologist.

---

2. *Venous Pulsation.* Dr. Breyer, (Journal Complement. Juin) relates the case of a young soldier, 26 years of age, who, having enjoyed previous good health, was seized with hemiplegia of the right side, attended with symptoms of cerebral congestion, and a general increased impetus of the circulation, the intellectual functions being sound. After several days, a universal pulsation of the veins was remarked, the arterial pulse at the wrist being full, hard, and incompressible, at 86 in the minute. The venous pulsations were perfectly synchronous with those of the heart and arteries. The whole surface appeared to rise and fall in correspondence with these pulsations—which were visible even in the eyes and tongue. These phenomena continued five days, and then disappeared. On the same evening the patient became comatose. On the sixth day from this time the venous pulsation again returned, but the patient still continued insensible. The pulsation subsided on the second day from this last period, and in two days more the patient died. Dissection threw no light on the cause of the venous pulsation.

Every practitioner must have noticed the blood flowing in jets from a vein, but we do not remember to have seen so unequivocal a proof of actual venous pulsation as the present case presents. It will hardly be argued that in this case the influence of the heart's action ceased when the blood arrived at the capillary vessels. The fact above narrated, without any theory in view, is a strong support to the doctrine of the heart being the great moving power of the circulation throughout the whole circle from the left ventricle to the right auricle. There can be no doubt, however, that the tonic and elastic power of the arteries acts as an important auxiliary to the heart under all circumstances—and even in the case just detailed.

---

3. *Hearing and Speech Restored.** We take the following particulars from the report made by the Institute, respecting this curious case. Honoré Trezel, nine years of age when operated on, was born at Paris, and was classed among those of the deaf and dumb, who have not the slightest sense of audition, even when close to the most violent explosions. His forehead was large, and his head well formed, but there was little expression in his countenance. He walked unsteadily, and he made known his more immediate wants by certain signs. These circumstances gave reason to believe that the imperfection was not accompanied by idiotism (as is too often the case) and consequently that an operation might not be useless. The operation was neither new nor difficult. It consisted in the introduction of injections into the eustachian canal by means of a small flexible tube—which injections were not followed, as is sometimes the case, by severe pains and fainting, nor by suppurations in the interior of the ear which destroy the good effects of the operation. The first few days after the restoration of hearing were, for young Honorè, a period of rapture—" un temps de ravissement." Every kind of noise (happy fellow) was to him like the music of the spheres! A musical snuff-box set him in ecstacy whenever it came within the reach of his auditory nerves; but he was some time in discovering that speech was a medium of communication between individuals. Even when he found that this was the case, he attached more importance to the movements of the lips than to the intonations of the voice and pronunciation of words. Hence he thought infants, when crying, were talking away at a most furious rate. He soon corrected this error, however; but unfortunately he heard a parrot chatter some phrases, and immediately generalising upon this datum (as older folks sometimes do in more important matters) he concluded that all animals had the faculty of speech, and addressed himself to them accordingly. He worked hard to make a dog pronounce the words *papa* and *pain*—the only words he had then learnt himself; but, as may be supposed, without success.

The restoration of audition produced a great change in the physical constitution of the boy. His gait became steady—his countenance brightened up and became more intellectual. But the power of speaking was very slow in being acquired, and it was long before he could recognise the direction of sounds which struck his ear. At length he was brought to pronounce some words of more than one syllable—and now his vanity knew no bounds. He scorned the society of the deaf and

---

* L'Oüie et la Parole rendues à Honoré Trezel, &c. Par le Docteur Deleau Jaun. 8vo. Paris, Sept. 1825.

dumb, among whom he had been placed, and considered himself quite on a level with other boys of his own age in general. So early does vanity take possession of the human mind—and so easily is it fanned into open flame!

Trezel made but a slow progress; yet, in the course of a year, from being completely deaf, he can now distinguish all kinds of sounds—evades carriages and horses—opens the door when he hears it rapped on—can appreciate music, and takes great pleasure in it, both vocal and instrumental—endeavours to imitate the modulations of voice which he hears, though with little success—can repeat all the words of the French language which are spoken before him—repeats by heart several phrases, &c. In short, there is reason to hope that this youth will be ultimately placed on a level with those of his age, and restored to that intercourse with society, from which he was cut off by the privation of an important sense.

The operation consisted in clearing the eustachian tube and admitting air into the ear.

